# Impact Modification of Poly(3-hydroxybutyrate) and Poly(3-hydroxybutyrate-*co*-3-hydroxyhexanoate) with Terratek FX1515 and Terratek GDH-B1FA

**DOI:** 10.1021/acsomega.5c01068

**Published:** 2025-05-27

**Authors:** Kush G. Patel, Adaeze R. Osakwe, Austin F. Wright, Virginia L. Weber, Huiming Wu, Shawn M. Wallbillich, Michael V. Kandefer, Grant H. Crane, Evan M. White, Jason J. Locklin

**Affiliations:** † School of Chemical, Materials, and Biomedical Engineering, College of Engineering, University of Georgia, Athens, Georgia 30602, United States; ‡ Department of Chemistry, Franklin College of Arts and Sciences, 1355 University of Georgia, Athens, Georgia 30602, United States; § New Materials Institute, University of Georgia, Athens, Georgia 30602, United States

## Abstract

Poly­(3-hydroxybutyrate) (PHB) and poly­(3-hydroxybutyrate-*co*-3-hydroxyhexanoate) (PHB-*co*-HHx) are promising alternatives to polyolefins due to their similar processing temperatures and mechanical properties. However, these polymers exhibit brittle failure and poor impact performance, limiting their uses for certain applications. In this work, industrially compostable impact modifiers Terratek FX1515 (FX1515) and Terratek GDH-B1FA (GDH-B1FA) were blended with PHB and PHB-*co*-HHx at 10, 20, and 30% w/w loadings to assess the mechanical performance of these polymers. Blends containing both impact modifiers show remarkable improvement in the tensile and Izod impact properties of both polymers. The impact strength of PHB-*co*-HHx blends increases from 2.50 ± 0.09 to 13.81 ± 1.91 kJ/m^2^ (30% FX1515) and 59.23 ± 1.27 kJ/m^2^ (30% GDH-B1FA), whereas PHB blends showed moderate improvement from 2.37 ± 0.07 to 4.07 ± 0.18 kJ/m^2^ (30% FX1515) and 4.79 ± 0.46 kJ/m^2^ (30% GDH-B1FA). Further analysis of the impact surfaces using scanning electron microscopy, dynamic mechanical analysis, and interfacial tension revealed that the better performance of GDH-B1FA blends was due to its inherent elastomeric properties, large domain size, and good compatibility at the polymer–polymer interface. Additionally, biodegradation testing of the neat polymers, impact modifiers, and blends (ASTM D5338, industrial composting) shows that the polymers and impact-modified blends are capable of composting fully within 120 days.

## Graphical abstract

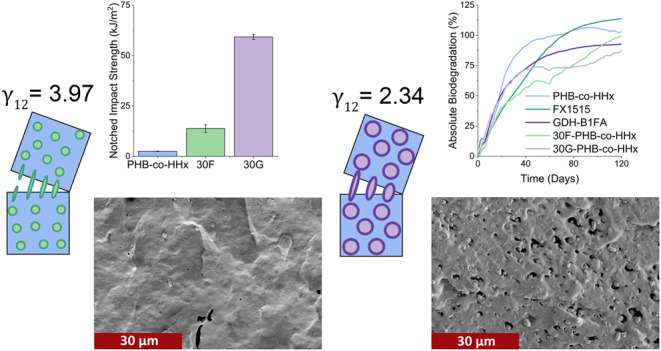

## Introduction

Poly­(hydroxyalkanoates) (PHAs) are a promising class of polymers capable of replacing conventional plastics, especially for single-use applications.^1,2^ PHAs have garnered attention in part due to their “greener” production process and their ability to biodegrade under many different environmental conditions.^3–6^ Additionally, by altering the comonomer as well as the comonomer ratio, PHAs can be tailored to have a broad range of mechanical properties ranging from flexible, low-density polyethylene-like (LDPE) properties to rigid polypropylene-like (PP) and even polystyrene-like (PS) properties.^6–9^ Over 150 comonomers of PHAs have been identified; among them, copolymers of poly­(3-hydroxybutyrate) (PHB) with 3-hydroxyvalerate (HV), 3-hydroxyhexanoate (HHx), and 4-hydroxybutyrate (4HB) have been recently commercialized.^4–7,9^

Unfortunately, PHB suffers from several limitations that affect its processability and mechanical performance. PHB is a highly crystalline and brittle polymer, largely due to its slow nucleation rate and the large spherulite size.^7,9–11^ Thermal degradation of PHB during melt-processing is also a significant issue, being that the melt temperature (*T*_m_) and degradation temperature overlap.^7,12,13^ Copolymerizing PHB with other monomers results in polymers with lower melting temperatures, which can then be processed at temperatures well below the degradation temperature.^3,6–9^ However, the addition of comonomers further exacerbates the already low nucleation efficiency of PHB, resulting in secondary crystallization of the polymer as it ages.^11,14^ For these reasons, PHAs with low comonomer content are primarily investigated for commercial applications, despite limited mechanical performance and thermal instability.^4^ Much like PHB, low-comonomer-ratio PHAs are also characterized as rigid polymers with low flexibility and low toughness.^6–9^

Conventional high-impact polymers such as high-impact polystyrene (HIPS) and acrylonitrile-butadiene-styrene (ABS) are produced by block copolymerization of rigid and elastomeric monomers.^15,16^ In these polymers, large rubbery blocks produce soft immiscible domains in a stiff polymer matrix. These soft, phase-separated domains dampen and dissipate mechanical stresses imparted on the polymer.^16^ Synthetic production of PHB-based block copolymers can be conducted through ring-opening polymerization of β-butyrolactone or 4,8-dimethyldioxocane-2,6-dione monomers with a flexible monomer such as ε-caprolactone.^17–19^ However, the high costs of producing these monomers make it impractical to scale such block copolymers for commercial applications. As an alternative, additives such as plasticizers and impact modifier resins are typically blended into these polymers to increase their toughness and flexibility.^15^

Plasticizers are miscible additives that impart flexibility to a polymer by reducing the glass transition temperature (*T*_g_) of the polymer and increasing the elongation at break of polymers.^15,20–22^ Impact modifiers, on the other hand, are low-*T*_g_ immiscible polymers which create soft domains in polymer matrix such as impact-modified PP or polycarbonate.^16,22–25^ Like the elastomer blocks in HIPS and ABS, the soft impact modifier domains dampen mechanical stresses from the bulk polymer matrix.^16,22^ However, unlike block copolymers, blends of most polymers with impact-modifying agents typically have poor interfacial adhesion, which leads to lower overall performance.^22,26–28^ The interfacial adhesion can be significantly improved by incorporating compatibilizing agents and reactive extrusion of the polymers.^22,26–33^ Commercially available impact modifiers are typically produced using polymers such as rubbers, thermoplastic elastomers (TPE), and thermoplastic polyurethanes (TPU).

The impact modification of rigid bioplastics such as PLA and PHAs with flexible biobased and biodegradable plastics has been previously investigated. PBAT,^27,29,34^ PBSA,^31,35,36^ PBS,^32,33^ PCL,^30,37–39^ and others^40,41^ have been blended with PLA and PHAs to improve their impact properties. Nonbiodegradable impact modifiers such as acrylic rubber (Metablen Type W (Mitsubishi Chemical Co. (MCC)), Blendex SS308 and SS350 (Galata Chemical (Galata))), methyl methacrylate-butadiene-styrene (MBS) rubber (Dow Paraloid, Metablen Type C and E), silicone-acrylic rubber (Metablen Type S), ABS (Blendex 362), and acrylonitrile-styrene-acrylic (ASA) (Blendex 960A) have been commercially advertised as additives for PLA.^42–44^ However, these resins are nonbiodegradable and their use in formulations can significantly impact the end-of-life fate of the final product.

To explore alternatives, the industry has started to produce impact modifier resins capable of biodegradation under industrial composting conditions. For example, Green Dot Bioplastics, Inc. produces a series of industrially compostable (ASTM D6400 and EN 13432–08) elastomer resins marketed under the Terratek Flex trademark.^45^ The resins are composed of thermoplastic starch, polyesters, and other biodegradable additives to produce different grades of elastomers with a wide range of physical and thermal properties. These elastomer resins are marketed for use in PLA formulations to improve their flexibility and toughness. Specifically, the Terratek GDH-B1FA resin has been shown to increase the notched and unnotched Izod properties of PLA to greater than 400%. The remarkable improvement of the impact performance of PLA with Terratek resins, combined with their biodegradable nature, makes them a promising candidate for the impact modification of PHAs.^45^ In this study, poly­(3-hydroxybutyrate) and poly­(3-hydroxybutyrate-*co*-3-hydroxyhexanoate) with Terratek GDH-B1FA and Terratek FX1515 resins are blended at various loadings to evaluate their effects on the mechanical properties of PHAs.

## Experimental Methods

### Materials

Poly­(3-hydroxybutyrate) (PHB, <1 mol % HHx, *M*_w_ = 507 kDa, *Đ* = 3.2) and poly­(3-hydroxybutyrate-*co*-3-hydroxyhexanoate) (PHB-*co*-HHx, 7 mol % HHx, *M*_w_ = 1170 kDa, *Đ* = 2.7) were produced at the New Materials Institute at the University of Georgia. Impact modifier resins Terratek FX1515 (referred to as FX1515 or F- for blends) and Terratek GDH-B1FA (referred to as GDH-B1FA or G- for blends) were kindly donated by Green Dot Bioplastics, Inc. All resins were dried under vacuum at 70 °C for at least 72 h prior to extrusion. Solvents used for analysis were of ACS reagent grade or higher in purity. All materials were used as received without further purification.

### Differential Scanning Calorimetry

Differential scanning calorimetry (DSC) analysis of polymer samples was conducted using a TA Instruments DSC 250 equipped with an RCS90 cooling system (TA Instruments). PHB samples (4–7 mg) were heated from room temperature to 200 °C at 10 °C/min followed by a cooling step to −80 °C at 10 °C/min. The sample is heated back to 210 °C at 10 °C/min and cooled again to −80 °C at 10 °C/min. PHB-*co*-HHx samples were analyzed using the same procedure with the first peak temperature of 180 °C and the second peak temperature of 200 °C. Terratek resins were analyzed with the same procedure as well with a peak temperature of 200 °C for both heating steps. The DSC thermograms were analyzed using TRIOS software to obtain the glass transition temperature, melting temperature, and % crystallinity (Δ*H*_0,PHA_ = 146 J/g).

### Polymer Processing

Neat PHAs, impact modifiers, and formulations containing 10, 20, and 30 w/w% impact modifiers were melt-blended using a Process 11 (Thermo Fisher) twin screw extruder with all zones except for the feed set at 150 °C (PHB-*co*-HHx samples and Terratek resins) or 175 °C (PHB samples) with a screw speed of 100 rpm. The extrudate was cut into pellets and flushed through a HAAKE Minilab II conical twin screw extruder at the same conditions and collected for injection molding. The samples were injection-molded by using a HAAKE Minilab II ram injection molder (Thermo Fisher). The samples were injection-molded into a 45 °C mold at 650 bar to produce tensile specimens (ASTM D638 Type V) and Izod/DMA specimens (ASTM D256, 3.1 mm × 12.6 mm × 64.0 mm).

### Tensile Testing

Tensile testing was conducted in accordance with ASTM D638 using a Shimadzu Autograph AGS-X (Shimadzu) equipped with a 1 kN load cell. The specimens were aged for 3 days before being tested. The test was conducted at 10 mm/min, and data analysis was conducted using Shimadzu Trapezium software. The data reported are the statistical average of 3 samples.

### Izod Impact Testing

Impact testing of polymer samples was conducted in accordance with ASTM D256 using a Tinius Olsen model 104 impact tester. The injection-molded polymer samples were aged for 3 days prior to testing, and the data reported is the statistical average of 3 samples.

### Scanning Electron Microscopy

Scanning electron microscopy (SEM) of cryo-fractured and impact-fractured surfaces was conducted using FE-SEM Thermo Fisher Teneo at Georgia Electron Microscopy. The cryo-fractured surfaces were prepared from an Izod specimen by submerging and breaking the samples in liquid nitrogen. The cryo-fractured surfaces were then sliced and adhered to SEM pucks. Impact-fractured samples were prepared by slicing the surface off of a broken Izod specimen and mounted on SEM pucks. The sample surfaces were sputter-coated with a 16 nm thick layer of Au/Pd, and the images were taken with an electron beam energy of 5.00 kV and 0.20 nA for investigating surface morphology.

### Dynamic Mechanical Analysis

Dynamic mechanical analysis (DMA) of polymer bars was conducted using a TA Instruments DMA Q800 instrument equipped with a nitrogen purge cooler. Samples aged 3 days were analyzed from −80 to 100 °C with an oscillation strain of 0.05% and 1 Hz frequency. The data generated was analyzed with TA Universal Analysis software.

### Film Preparation

Films of polymers and impact modifiers were melt-pressed using a Carver model 4386 hydraulic press system. The polymer was placed between heated platens for 30 s at 6 tons of pressure at the extrusion temperatures provided above. The film was removed from the press and aged for 1 week at room temperature before testing.

### Surface Energy and Interface Tension Analysis

Contact angle measurements were carried out using Kruss DSA 100E (DE) onto prepared films using the sessile drop method. Measurements were recorded and analyzed at room temperature with the Drop Shape Analyzer image analysis software (Krüss GmbH, DE). Solvent drops (10 μL) of DI water, ethylene glycol, ethylene glycol/water (50:50 v/v), benzyl alcohol, and cyclohexane with known surface tensions (Table S2) were dispensed using a motor-driven syringe. The contact angle values were reported as the average of five drops on different points per sample. The surface energy (γ) was determined by the average contact angles of the five different test liquids and their respective free energies using the Owens–Wendt–Rabel–Kaelble (OWRK) model. Using the overall surface energy value (γ) and its individual components (dispersive (γ*^d^*) and polar (γ*^p^*)), the interfacial tension (γ_12_) between the polymers was calculated using the method described by Wu^46^ (eq 1).

1 $$\gamma_{12}=\gamma_{1}+\gamma_{2}-\frac{4\gamma_{1}^{d}\gamma_{2}^{d}}{\gamma_{1}^{d}+\gamma_{2}^{d}}-\frac{4\gamma_{1}^{p}\gamma_{2}^{p}}{\gamma_{1}^{p}+\gamma_{2}^{p}}$$

### Biodegradation Studies

The biodegradation analyses of the Terratek GDH-B1FA- and Terratek FX1515-modified PHB samples were performed with an ECHO Instruments (SI) Automated Respirometer System (with ±10% precision), in accordance with the testing standards outlined in ASTM D5338–15 (modified for automated respirometry) and ASTM 6400–19 (for verification of test material biodegradability). Carbon mineralization calculations were made by comparing the metabolic CO_2_ production rates of testing material channels to control channels containing blank inoculums (negative controls) and cellulose-loaded inoculums (positive controls). Testing materials were cryo-milled before introduction to sealed bioreactors in triplicate, prefilled with compost inoculum that passed the validating criteria outlined in ASTM D5338–15 for the general quality of the inoculum. Each reactor possessed a lower reservoir of deionized water for moisture retention of the inoculum through evaporation.

Tests and controls were incubated at 58 ± 2 °C for 120 days. Contents of all reactors were remoisturized and turned weekly. Atmospheric air was pumped through the respirometer to each channel via a multistep, water and air filtration system at a rate of approximately 200 mL per minute. Before analyzing the atmospheric gas composition of each channel via an infrared spectrometer cell, water was removed from the air via a condensation trap and returned back to the reactors. Calculation methods for biodegradation are provided in the Supporting Information.

## Results and Discussion

### Mechanical Properties

Tensile properties of both PHAs and their blends with impact modifiers are shown in Figure 1 and listed in Table 1. As expected, after 3 days of aging, the homopolymer PHB had much higher elastic modulus (1.50 ± 0.02 GPa) and lower strain at break (5.3 ± 0.8%ε) than the copolymer PHB-*co*-HHx (0.89 ± 0.04 GPa and 18.9 ± 2.1%ε). However, the impact of Terratek resins on the %ε was significantly lower in PHB than PHB-*co*-HHx, as shown in Figure 1 and Table 1. The tensile properties of F-PHB samples were also different than those of G-PHB samples. 10F-PHB, 20F-PHB, and 30F-PHB all have higher modulus and lower strain at break compared to G-PHB samples, which had lower modulus and much higher elongation (Figure 1c,d, and Table 1). In fact, 30G-PHB samples show a high %ε (44.01 ± 4.13%), which was unexpected of a rigid polymer such as PHB (Figure 1d and Table 1).

**Figure 1:**
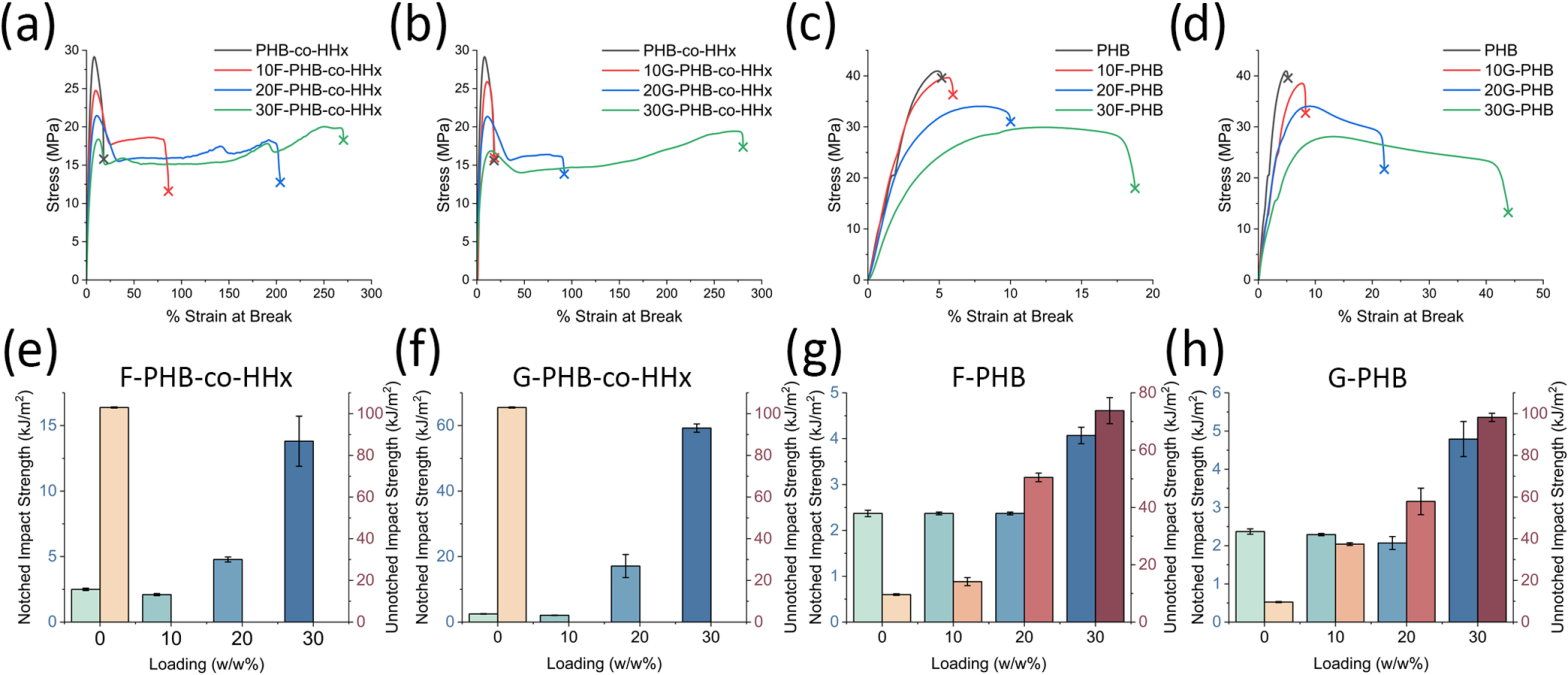
Mechanical performance of PHAs and their blends with Terratek resins. (a) Tensile properties of F-PHB-*co*-HHx. (b) Tensile properties of G-PHB-*co*-HHx. (c) Tensile properties of F-PHB. (d) Tensile properties of G-PHB. (e) Impact properties of F-PHB-*co*-HHx. (f) Impact properties G-PHB-*co*-HHx. (g) Impact properties F-PHB. (h) Impact properties G-PHB. Blue bars depict notched Izod values, and red bars depict unnotched Izod values.

**Table 1:** Mechanical Properties of PHAs and Their Blends with Terratek Resins. Commercial PP values were obtained from a technical datasheet for ExxonMobil PP1013H1 resin published by Exxon Mobil Corporation^47^

sample	Young’s modulus (GPa)	% elongation at break	yield stress (MPa)	notched impact strength (kJ/m^2^)	unnotched impact strength (kJ/m^2^)
commercial PP^47^	1.50 ^47^		33.5 ^47^	3.1 ^47^	
PHB	1.50 ± 0.02	5.3 ± 0.8	16.18 ± 2.85	2.37 ± 0.07	9.61 ± 0.29
PHB-*co*-HHx	0.89 ± 0.04	18.9 ± 2.1	14.80 ± 0.72	2.50 ± 0.09	102.97 ± 0.33
FX1515	0.058 ± 0.001	468.5 ± 11.9	5.59 ± 0.03	-	
GDH-B1FA	0.016 ± 0.0002	701.9 ± 15.6	2.57 ± 0.02	-	
10F-PHB	1.35 ± 0.06	6.9 ± 0.4	27.49 ± 0.42	2.37 ± 0.03	14.12 ± 1.40
20F-PHB	1.13 ± 0.05	11.5 ± 1.6	21.03 ± 1.83	2.37 ± 0.03	50.49 ± 1.51
30F-PHB	0.88 ± 0.07	19.3 ± 1.6	15.69 ± 2.07	4.07 ± 0.18	73.79 ± 4.56
10G-PHB	1.06 ± 0.02	8.9 ± 1.1	10.26 ± 0.67	2.29 ± 0.03	37.14 ± 0.65
20G-PHB	1.04 ± 0.06	22.0 ± 1.4	11.06 ± 1.09	2.07 ± 0.17	57.90 ± 6.36
30G-PHB	0.82 ± 0.09	44.01 ± 4.13	11.11 ± 3.83	4.79 ± 0.46	98.23 ± 1.97
10F-PHB-*co*-HHx	0.72 ± 0.01	87.3 ± 10.7	11.5 ± 0.05	2.10 ± 0.08	no break
20F-PHB-*co*-HHx	0.61 ± 0.02	201.0 ± 8.6	9.92 ± 0.18	4.78 ± 0.19	no break
30F-PHB-*co*-HHx	0.45 ± 0.01	271.7 ± 10.3	8.04 ± 0.06	13.81 ± 1.91	no break
10G-PHB-*co*-HHx	0.70 ± 0.05	17.4 ± 2.1	13.19 ± 0.88	2.07 ± 0.08	no break
20G-PHB-*co*-HHx	0.64 ± 0.04	93.3 ± 7.9	10.04 ± 0.08	17.11 ± 3.54	no break
30G-PHB-*co*-HHx	0.43 ± 0.01	279.8 ± 5.0	7.65 ± 0.15	59.23 ± 1.27	no break

The tensile performance of F-PHB-*co*-HHx and G-PHB-*co*-HHx blends followed an opposite trend from PHB samples (Figure 1 and Table 1). The elastic modulus values were similar for both impact modifier blends, but the strain at break for the GDH-B1FA samples was much lower for 10G-PHB-*co*-HHx and 20G-PHB-*co*-HHx compared to those for 10F-PHB-*co*-HHx and 20F-PHB-*co*-HHx (Figure 1a,b and Table 1). The only exception was 30G-PHB-*co*-HHx, which performed similarly to the 30F-PHB-*co*-HHx samples in both modulus and %ε.

However, the improvement in impact properties of PHB-*co*-HHx blends was drastically different from those observed in tensile testing. The impact performance of G-PHB-*co*-HHx blends was significantly higher than that of F-PHB-*co*-HHx. Compared to control PHB-*co*-HHx (2.50 ± 0.09 kJ/m^2^), 30F-PHB-*co*-HHx (13.81 ± 1.91 kJ/m^2^, >550%), 20G-PHB-*co*-HHx (17.11 ± 3.54 kJ/m^2^, >680%), and 30G-PHB-*co*-HHx (59.23 ± 1.27 kJ/m^2^, >2350%) all showed remarkable improvement in impact properties (Figure 1e,f and Table 1). The 10% impact modifier-loaded samples performed slightly worse than the control samples in all cases, likely due to the lack of impact modifier domains to dissipate the applied stress. The unnotched samples of PHB-*co*-HHx blended with both impact modifiers did not break fully at the highest force tested (11.77 N pendulum).

The notched Izod of PHB (2.37 ± 0.07 kJ/m^2^) was comparable to PHB-*co*-HHx (2.50 ± 0.09 kJ/m^2^) and commercial PP (ExxonMobil PP1013H1)^47^ resin (3.1 kJ/m^2^). However, the inherent differences between the two polymers were revealed with unnotched Izod values. PHB-*co*-HHx (102.97 ± 0.33 kJ/m^2^) performed over an order of magnitude higher than PHB (9.61 ± 0.29 kJ/m^2^), revealing that the copolymer is inherently tougher (Figure 1 and Table 1). The primary reason for the poorer performance of PHB was the high degree of crystallinity. As shown in Figure S1 and Table S1, the PHB samples were all >70% crystalline, whereas the crystallinity of PHB-*co*-HHx samples was 40–50%. Higher crystallinity results in a much lower flexibility of PHB and reduces the ability of the polymer to dissipate applied stress. PHB is also well known to form large spherulites, which can be detrimental to mechanical properties.^9,22^

Additionally, the notched Izod of the impact-modified PHB samples did not show any improvement until 30% loading. On the other hand, PHB-*co*-HHx samples show remarkable improvement at 20% loading (Figure 1e–h and Table 1). The unnotched Izod of PHB did show significant improvement with the impact strength at all % loadings with 30F-PHB (73.79 ± 4.56 kJ/m^2^) and 30G-PHB (98.23 ± 1.97 kJ/m^2^). This was >760 and >1020% greater than neat PHB (Figure 1g,h and Table 1), respectively. Drastic improvement in unnotched samples with and without impact modifiers was attributed to the pseudoductile nature of these blends. This means that the PHA blends possess high crack initiation resistance but poor crack propagation resistance, which results in high unnotched Izod performance relative to notched Izod performance.^48^

In both polymers, the significant differences in the impact performance between FX1515 and GDH-B1FA can be attributed to the more elastomer-like properties of GDH-B1FA. As observed in the tensile properties (Table 1), GDH-B1FA has a lower modulus (0.016 ± 0.0002 GPa) and higher strain at break (701.9 ± 15.6%ε) compared to FX1515 (0.058 ± 0.001 GPa and 468.5 ± 11.9%ε), which likely allows the material to dissipate the applied stress with higher efficiency.

### Morphology

Scanning electron microscopy (SEM) images of the cryo- and impact-fractured specimens were taken to further understand the interfacial adhesion between the impact modifiers and PHAs. The SEM images shown in Figures 2 and S2–S5 reveal that F-PHB and G-PHB undergo minimal deformation in both the cryo- and impact-fractured surfaces. Submicron voids in control PHB samples were observed, which can be attributed to the presence of PHB granules that were not melted during the extrusion process. As seen in the DSC thermograms in Figure S1 and data from Table S1, the *T*_m_ of neat PHB was 176 °C and the melt end-set was higher than the extrusion temperature of 175 °C. The poor performance of the 10 and 20% impact modifier-loaded samples was attributed to the smaller impact modifier domain size in the bulk PHB matrix (Figures S2–S5). By comparison, samples with higher impact modifier loading of 30% had a greater prevalence of impact modifier domains dispersed throughout the matrix, which dissipate the impact energy more efficiently (Figures 2 and S2–S5).

**Figure 2:**
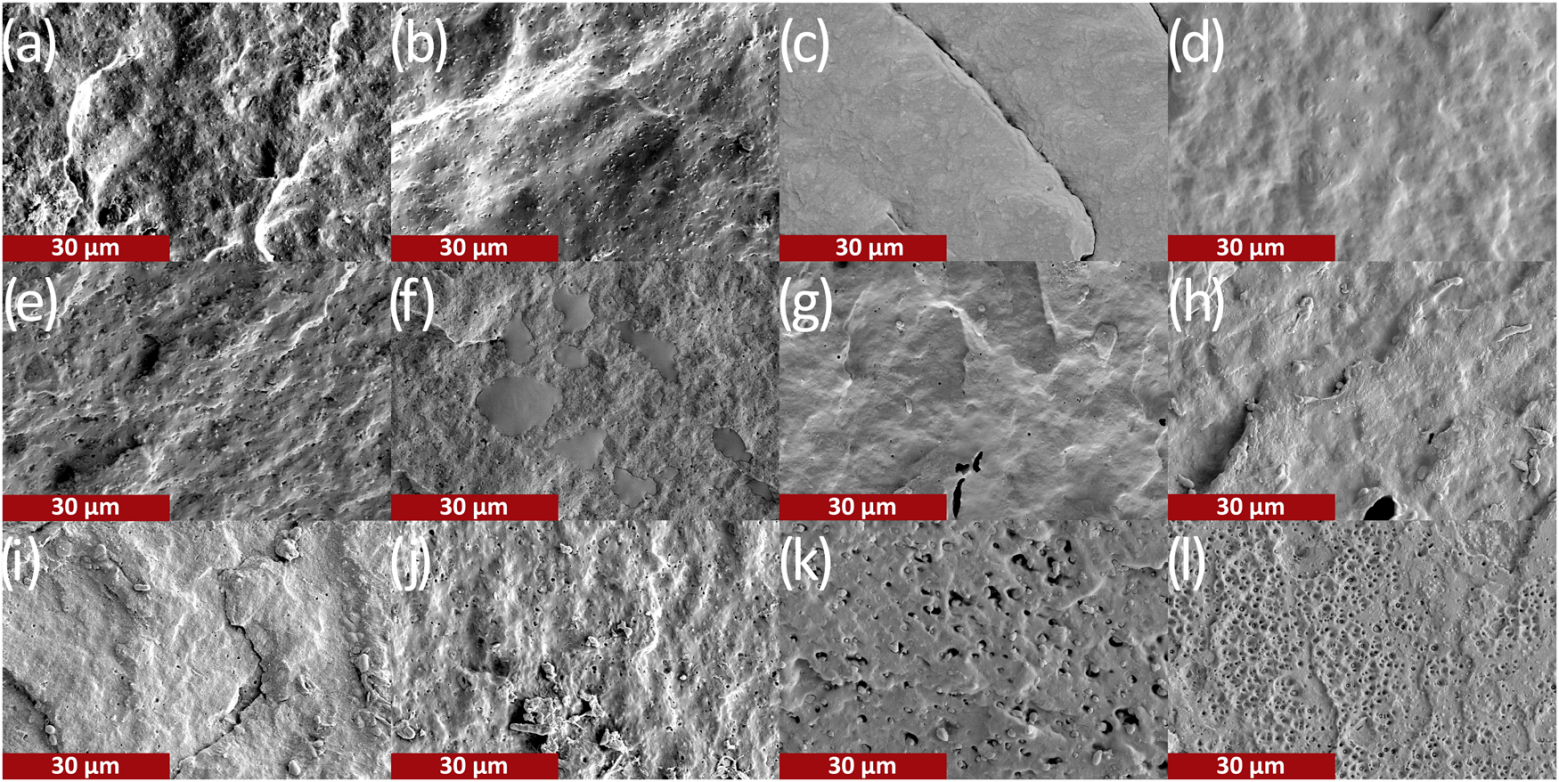
Scanning electron microscopy images of PHAs and their blends with Terratek resins. (a) PHB cryo-fractured; (b) PHB impact-fractured; (c) PHB-*co*-HHx cryo-fractured; (d) PHB-*co*-HHx impact-fractured; (e) 30F-PHB cryo-fractured; (f) 30F-PHB impact-fractured; (g) 30F-PHB-*co*-HHx cryo-fractured; (h) 30F-PHB-*co*-HHx impact-fractured; (i) 30G-PHB cryo-fractured; (j) 30G-PHB impact-fractured; (k) 30G-PHB-*co*-HHx cryo-fractured; and (l) 30G-PHB-*co*-HHx impact-fractured.

PHB-*co*-HHx samples were observed to have drastic differences between the cryo- and impact-fractured samples. The cryo-fractured samples reveal that the FX1515 samples were dispersed through the PHB-*co*-HHx matrix in much smaller phase-separated domains, indicating good miscibility between the two materials in 10F, 20F, and 30F samples (Figures 2g and S6). On the other hand, the impact-fractured surface showed that some of the impact modifier domains had deformed, elongated into strands, and left behind cavities on the surface of the 20F and 30F blends (Figures 2h and S7). The extent of deformation indicates that there is good adhesion at the polymer-impact modifier interface.

The 10G-PHB-*co*-HHx samples had similar performance and phase morphology to 10F-PHB-*co*-HHx samples for cryo- and impact-fractured surfaces (Figures S6–S9). However, at higher loadings, larger domains in the cryo-fractured surfaces were observed, especially for the 30G sample, indicating poorer miscibility between the two materials (Figure S8). At the same time, the deformation pattern observed between the 20G and 30G impact-fractured surfaces indicates that, despite the poor miscibility between the two materials, strong interfacial adhesion was present (Figure S9). The 20G-PHB-*co*-HHx Izod sample had impact modifier domains slightly elongated and protruding from the surface of the bulk matrix in a similar fashion as the 30F-PHB-*co*-HHx sample, but to a lower extent (Figures S7 and S9). Unlike 20G-PHB-*co*-HHx and 30F-PHB-*co*-HHx, the impact-fractured surface of 30G-PHB-*co*-HHx revealed that the surface was deformed significantly and covered with craters of the impact modifier (Figure S9). The larger impact modifier domains and the higher degree of deformation observed between GDH-B1FA and PHB-*co*-HHx are inferred to be the causes of the significant improvements in impact performance. Additionally, the inherent mechanical properties of GDH-B1FA (lower modulus and higher % strain at break) appeared to have a crucial role in the drastic improvement of the impact performance, as compared with FX1515 (Table 1).

### Thermomechanical Properties

Polymer miscibility can be investigated by using dynamic mechanical analysis (DMA) and DSC to observe changes in the glass transition temperature of the blends. Polymers that are fully miscible with one another will result in a glass transition temperature relative to the weight fractions of the polymers and their respective glass transition temperatures.^15,22^ Most polymer blends, including those studied in this work, are clearly immiscible, as observed in the SEM images. However, even in such cases, partial miscibility can be observed by changes in the glass transition temperature of the blend. As seen in Figure S1 and Table S1, the glass transition temperatures of the PHA blends shift between the loss modulus peaks of the neat polymers.^22^

DMA thermograms of PHB-*co*-HHx samples loaded with FX1515 (Figure 3a) revealed a significant shift in the peak temperature of the loss modulus for both PHB-*co*-HHx and impact modifier. The loss modulus peak temperature of FX1515 increased by 22.4 °C at 10%, 21.1 °C at 20%, and 20.0 °C at 30% loading (Figure 3a and Table 2). The loss modulus peak temperature of PHB-*co*-HHx on the other hand steadily decreased by −4.9 °C (10% loading), −9.5 °C (20% loading), and −13.1 °C (30% loading) (Figure 3a and Table 2). These results indicate that a significant amount of impact modifier interacts with the amorphous domains of PHB-*co*-HHx, which in turn results in a smaller domain size and better tensile performance.

**Table 2:** Dynamic Mechanical Properties of PHB, PHB-*co*-HHx, Terratek Resins, and Blends

sample	*T*_g,impact modifier_^a^ (°C)	*T*_g,impact modifier_^b^ (°C)	*T*_g,PHA_^a^ (°C)	*T*_g,PHA_^b^ (°C)
PHB			10.8	20.8
PHB-*co*-HHx			7.2	14.6
10F-PHB-*co*-HHx	–27.6	–20.9	0.6	9.7
20F-PHB-*co*-HHx	–28.9	–20.7	–2.4	5.1
30F-PHB-*co*-HHx	–30.0	–22.8	–6.3	1.5
10G-PHB-*co*-HHx	–36.5	–30.1	2.6	12.6
20G-PHB-*co*-HHx	–35.4	–28.2	1.0	11.2
30G-PHB-*co*-HHx	–34.9	–27.9	–0.8	8.4
10F-PHB	–28.6	–19.8	6.2	16.9
20F-PHB	–30.6	–22.0	0.1	12.8
30F-PHB	–28.8	–22.5	–2.5	6.9
10G-PHB	–30.3	–21.9	8.0	18.9
20G-PHB	–32.8	–24.2	6.7	16.1
30G-PHB	–32.2	–24.5	3.5	16.0
Terratek FX1515	–50.0	–36.9		
Terratek GDH-B1FA	–47.8	–30.0		

a Obtained from the storage modulus onset.

b Obtained from the peak of loss modulus.

**Figure 3:**
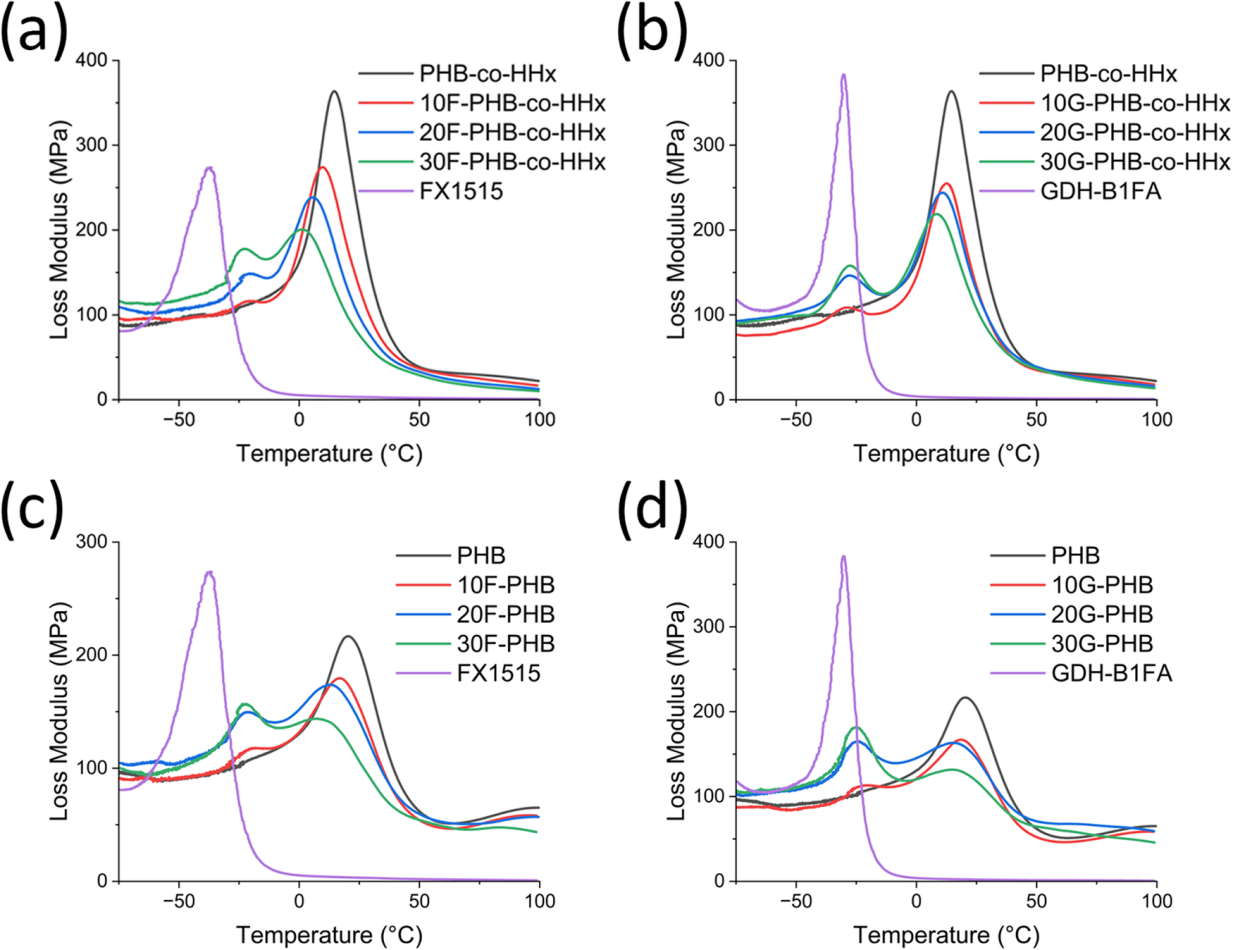
Loss modulus thermograms of PHAs, Terratek resins, and their blends. (a) F-PHB-*co*-HHx; (b) G-PHB-*co*-HHx; (c) F-PHB; and (d) G-PHB.

On the other hand, samples of PHB-*co*-HHx loaded with GDH-B1FA (Figure 3b) did not display as significant a change in the loss modulus peak temperature of either polymer. The loss modulus peak temperature of GDH-B1FA increased by 11.3 °C at 10%, 12.4 °C at 20%, and 12.9 °C at 30% loadings (Figure 3b and Table 2). The loss modulus peak temperature of PHB-*co*-HHx on the other hand steadily decreased by −2 °C (10% loading), −3.4 °C (20% loading), and −6.2 °C (30% loading) (Figure 3b and Table 2). These results suggest that the impact modifier GDH-B1FA has limited miscibility, which leads to a larger domain size in the blend. The excellent impact performance is likely due to larger impact modifier domains and good interfacial compatibility.

Much like PHB-*co*-HHx samples, PHB samples loaded with FX1515 (Figure 3c) show a significant change in the loss modulus peak of FX1515. Increases in loss modulus peak temperature of 21.4 °C at 10%, 19.4 °C at 20%, and 21.2 °C at 30% loading indicate partial miscibility between the two polymers (Table 2). A similar decrease in the loss modulus peak temperature was also observed in the GDH-B1FA (Figure 3d) samples. The loss modulus peak increased by 17.5 °C at 10%, 15.0 °C at 20%, and 15.6 °C at 30% loading. This change in loss modulus peak temperature of G-PHB samples was lower compared to the F-PHB samples. These results indicate that G-PHB samples may have better miscibility than G-PHB-*co*-HHx blends but not to the same extent as F-PHB samples. Additionally, given that the GDH-B1FA domains in PHB were not significantly larger than FX1515 domains, the better performance of the GDH-B1FA samples can be attributed to the more elastomeric behavior of the impact modifier.

### Interface Tension

Mechanical performance of immiscible polymer blends is dependent on the interactions at the interface. Interfacial tension quantifies the extent of polymer–polymer interactions. Interfacial tension of polymer blends can be indirectly measured using the surface energy of the polymers using the method described by Wu.^46^ However, this method is heavily dependent on the quality of the surface energy values obtained. Surface energy value is often measured using contact angle methods, where various solvents of known free surface energy are tested on a polymer substrate. The method provides reliable surface energy values, but the solvents used must be carefully selected. The two-component model described by Owens, Wendt, Rabel, and Kaelble (OWRK) is the most used in the literature. Other methods such as Fowkes theory are difficult to apply to polymers that are moderately polar and easily swell in high-dispersity solvents such as diiodomethane. With the OWRK theory, diiodomethane can be replaced with other solvents that do not swell the polymer. Additionally, using solvents of high polarity, moderate polarity, and purely dispersive properties provides better characterization of the surface energy.^49^ The contact angle measurements and surface energy values for polymers and impact modifiers are given in Tables S3 and S4.

Generally, lower interfacial tension between two polymers indicates greater compatibility at the interface.^46,50,51^ As calculated from the surface energy, the interfacial tension between F-PHB was 1.74 mN/m, while G-PHB had a higher interfacial tension of 3.00 mN/m (Table 3). These results indicate the FX1515 likely had better adhesion with the bulk PHB matrix, which allowed for the less elastomeric impact modifier to perform comparably to G-PHB blends. On the other hand, G-PHB-*co*-HHx had lower interfacial tension (2.34 mN/m) compared to F-PHB-*co*-HHx (3.97 mN/m) (Table 3). This suggests that the drastic improvement in the impact performance was likely due to these strong interface interactions.

**Table 3:** Interfacial Tension (γ_12_) of Polymer Impact Modifier Blends

blends	γ_12_ (mN/m)
PHB/FX1515	1.74
PHB/GDH-B1FA	3.00
PHB-*co*-HHx/FX1515	3.97
PHB-*co*-HHx/GDH-B1FA	2.34

### Degradation Studies

In addition to the exceptional impact performance of Terratek-PHA blends, the end of life of these materials makes them valuable additives for products intended for industrial composting conditions. To better understand the effects of these resins on the biodegradation of PHAs, respirometry was conducted to simulate biodegradation under industrial composting conditions (ASTM 6400–19 and modified ASTM D5338–15). Samples with the highest loading of impact modifier were chosen for the study as extreme conditions for these blends. The results from the composting studies are shown in Figure 4 (absolute biodegradation).

**Figure 4:**
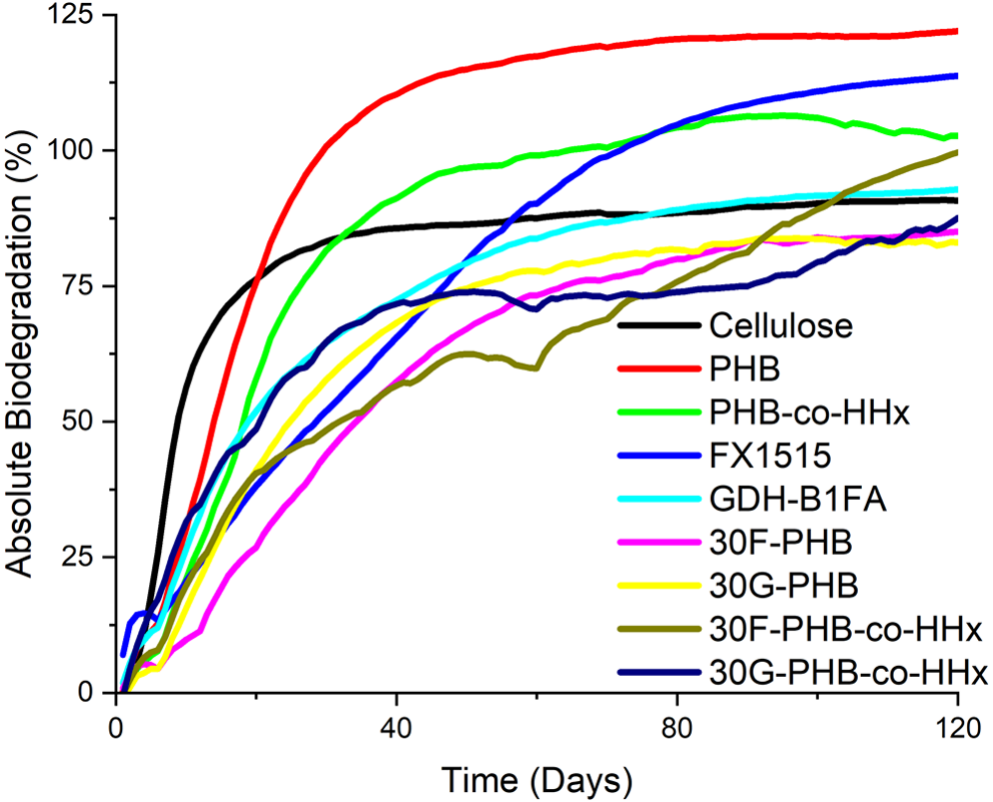
Absolute biodegradation profiles of PHAs, Terratek resins, PHA-Terratek blends, and cellulose were determined from CO_2_ evolution.

By day 45 of testing, the cellulose control reached 86% absolute biodegradation, indicating the validity of the test in accordance with ASTM D5338–15§13.2. The low observed mineralization of the cellulose control may be associated with a higher concentration of nitrates derived primarily from poultry bedding and manure inputs in the compost inoculum (Table S5), with a low but acceptable C/N ratio of 11.25. Because the cellulose controls did not produce 100% of the theoretical CO_2_ by the end of testing, samples may be evaluated relative to the positive control as per ASTM D6400–19§6.3.1 as shown in Figure S11 and summarized in Table S6.

Despite observing low biodegradation of the cellulose control in absolute, the neat PHAs were observed to pass 90% biodegradation by 25 days (PHB) and 38 days (PHB-*co*-HHx). The biodegradation rates of the impact modifier resins were observed to be lower than those of neat PHAs, but these resins reached 90% biodegradation by 59 days (FX1515) and 87 days (GDH-B1FA). Additionally, the neat PHAs and FX1515 samples exhibited biodegradability measurements that exceeded 100%. This overproduction of CO_2_ in the absence of excess organic carbon was attributed to a “priming” effect that has been observed to take place within various respirometry inoculums, which can result in acceleration or retardation of biodegradation of soil organic matter.^52–54^ This phenomenon has not been well documented with PHAs in prior literature but in the past has been attributed to starches and cellulose.^52,54,55^ At the end of the study, 30F-PHB, 30G-PHB, and 30G-PHB-*co*-HHx reached 93.7, 91.5, and 96.5% biodegradation relative to cellulose, respectively. All samples mineralized over 90% relative to cellulose (Table S6) and may be regarded as industrially compostable materials.

## Conclusions

Terratek FX1515 and Terratek GDH-B1FA significantly improved the tensile and impact performances of PHB and PHB-*co*-HHx. Blends of both polymers with GDH-B1FA performed much better at impact modification. In PHB samples, on the other hand, no improvement was observed at 10 and 20% loadings, and improvements of 150–200% were observed in notched samples. For PHB-*co*-HHx, the notched Izod performance of 30G-PHB-*co*-HHx samples was observed to be exceptional—over 2350% higher than control—while significant improvement of >550% was observed in 30F-PHB-*co*-HHx. The unnotched samples showed even greater improvement for both PHB and PHB-*co*-HHx samples, where PHB samples improved by 760–1020% at 30% impact modifier loadings and no break was achieved in the PHB-*co*-HHx samples loaded with impact modifier.

The underlying mechanism for the better performance of GDH-B1FA relative to FX1515 was attributed to the more elastomeric nature of the resin, lower interfacial tension, and lower miscibility. The latter resulted in much larger and more well-defined GDH-B1FA phases dispersed within the polymer matrix, which dissipated mechanical stresses more effectively than the smaller phases observed with FX1515. The interactions between the polymers and impact modifiers were evaluated by DMA, DSC, and surface energy measurements to understand the significant difference in the impact performance of PHB blends and PHB-*co*-HHx blends. DMA revealed that the samples containing FX1515 had a significant shift in the peak temperature of loss modulus in PHB and PHB-*co*-HHx, whereas the GDH-B1FA samples had a lower loss modulus peak shift. The interfacial tension between the polymer blends suggested that the less miscible GDH-B1FA has greater affinity with PHB-*co*-HHx than FX1515 at the interface, which likely resulted in the drastic improvement of impact performance. On the other hand, PHB has a greater affinity for the less elastomeric FX1515 than GDH-B1FA, which may have contributed to F-PHB blends having relatively close Izod performance to G-PHB blends. The respirometry study revealed that the blends and neat polymers are capable of mineralizing under industrial composting conditions with all resins achieving over 90% relative degradation by 120 days.

## Supplementary Material



## Notes

DSC thermograms of polymers and blends; SEM images of polymers and blends; DMA properties of polymers and blends; contact angle and surface energy values of solvents and polymers; elemental analysis of compost; and respirometry calculations and analysis (PDF)
