# Supplementary material for: Impact Modification of Poly(3-hydroxybutyrate) and Poly(3-hydroxybutyrate-co-3-hydroxyhexanoate) with Terratek FX1515 and Terratek GDH-B1FA
Source: ACS Omega. 2025 May 27;10(22):23025–34. doi: 10.1021/acsomega.5c01068 (PMC12163630; doi:10.1021/acsomega.5c01068)
Supplement: Supplementary file 1 [file ao5c01068_si_001.pdf]

Supporting Information for:

Impact Modification of Poly(3-hydroxybutyrate) and Poly(3-hydroxybutyrate-*co*-3-hydroxyhexanoate) with Terratek FX1515 and Terratek GDH-B1FA

Kush G. Patel[†,§], Adaeze R. Osakwe[‡,§], Austin F. Wright[§], Virginia L. Weber[‡,§], Huiming Wu[‡,§], Shawn M. Wallbillich[§], Michael V. Kandefer[§], Grant H. Crane[§], Evan M. White[§], and Jason J. Locklin[†,‡,§,*]

† School of Chemical, Materials, and Biomedical Engineering, College of Engineering, University of Georgia, Athens, GA 30602.

‡ Department of Chemistry, Franklin College of Arts and Sciences, University of Georgia, Athens, GA, 30602.

§ New Materials Institute, University of Georgia, Athens, GA, 30602.

* Corresponding Author – email: jlocklin@uga.edu

Number of pages: 10

Number of tables: 5

Number of figures: 11

Table of Contents

Table S1. Thermal properties of polymers, impact modifiers, and blends. **3**

Table S2. Surface tension of solvents used. **8**

Table S3. Contact angle measurements of PHAs and impact modifiers. **8**

Table S4. Surface energy of PHAs and impact modifiers. **9**

Table S5. Elemental analysis of compost used for respirometry. **10**

Table S6. Respirometry results of PHAs, blends, and impact modifiers. **10**

Figure S1. DSC thermograms of polymers, impact modifiers, and blends. **3**

Figure S2. SEM micrographs of cryo-fractured PHB/FX1515 blends. **4**

Figure S3. SEM micrographs of impact-fractured PHB/FX1515 blends. **4**

Figure S4. SEM micrographs of cryo-fractured PHB/GDH-B1FA blends. **5**

Figure S5. SEM micrographs of impact-fractured PHB/GDH-B1FA blends. **5**

Figure S6. SEM micrographs of cryo-fractured PHB-*co*-HHx/FX1515 blends. **6**

Figure S7. SEM micrographs of impact-fractured PHB-*co*-HHx/FX1515 blends. **6**

Figure S8. SEM micrographs of cryo-fractured PHB-*co*-HHx/GDH-B1FA blends. **7**

Figure S9. SEM micrographs of impact-fractured PHB-*co*-HHx/GDH-B1FA blends. **7**

Figure S10. Storage modulus thermograms of PHAs, blends, and impact modifiers. **8**

Figure S11. Respirometry results of PHAs, blends, and impact modifiers. **9**

Differential Scanning Calorimetry (DSC)

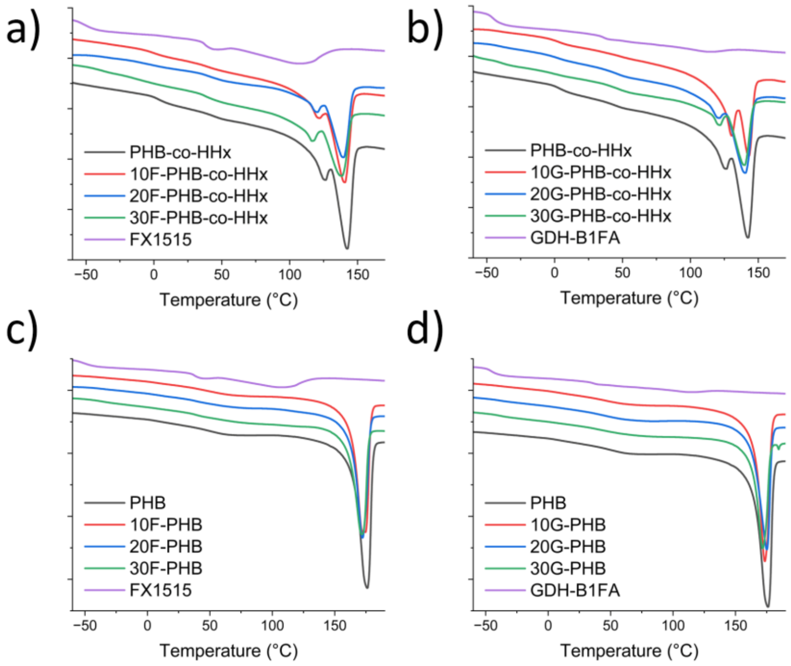

Figure S1. DSC thermograms of PHAs, Terratek resins, and their blends. a) PHB-*co*-HHx, Terratek FX1515, and their blends; b) PHB-*co*-HHx, Terratek GDH-B1FA, and their blends; c) PHB, Terratek FX1515, and their blends; and d) PHB, Terratek GDH-B1FA, and their blends. All plots show exothermic transitions up.

Table S1. Thermal properties of PHB, PHB-*co*-HHx, Terratek resins, and blends. (a – obtained from the first heating scan; b – obtained from the second heating scan; and N/O – not observed).

**Sample**	$T_{g,\text{Impact Modifier}}{}^{b}$ (˚C)	$T_{g,\text{PHA}}{}^{b}$ (˚C)	$T_{m,\text{Impact Modifier}}{}^{a}$ (˚C)	$T_{m,\text{PHA}}{}^{a}$ (˚C)	$\%X_{C,\text{PHA}}{}^{a}$
PHB	-	5.2	-	176.0	72.9
PHB-*co*-HHx	-	2.3	-	142.3	41.5
10F-PHB-*co*-HHx	N/O	-1.7	-	140.4	44.4
20F-PHB-*co*-HHx	-37.6	-5.2	-	139.5	49.5
30F-PHB-*co*-HHx	-38.5	-9.2	-	138.1	45.1
10G-PHB-*co*-HHx	N/O	2.1	-	142.9	41.7
20G-PHB-*co*-HHx	-40.2	1.4	-	140.1	50.1
30G-PHB-*co*-HHx	-38.1	0.0	-	139.7	52.7
10F-PHB	N/O	2.5	-	172.4	72.5
20F-PHB	-39.5	1.6	-	172.3	76.4
30F-PHB	-39.3	-3.7	-	171.7	81.0
10G-PHB	N/O	4.8	-	173.5	70.2
20G-PHB	-35.7	3.5	-	175.0	70.1
30G-PHB	-36.2	2.8	-	171.7	73.7
Terratek® FX1515	-50.3	-	105.8	-	-

Terratek® GDH-B1FA	-45.3	-	110.9	-	-

Scanning Electron Microscopy

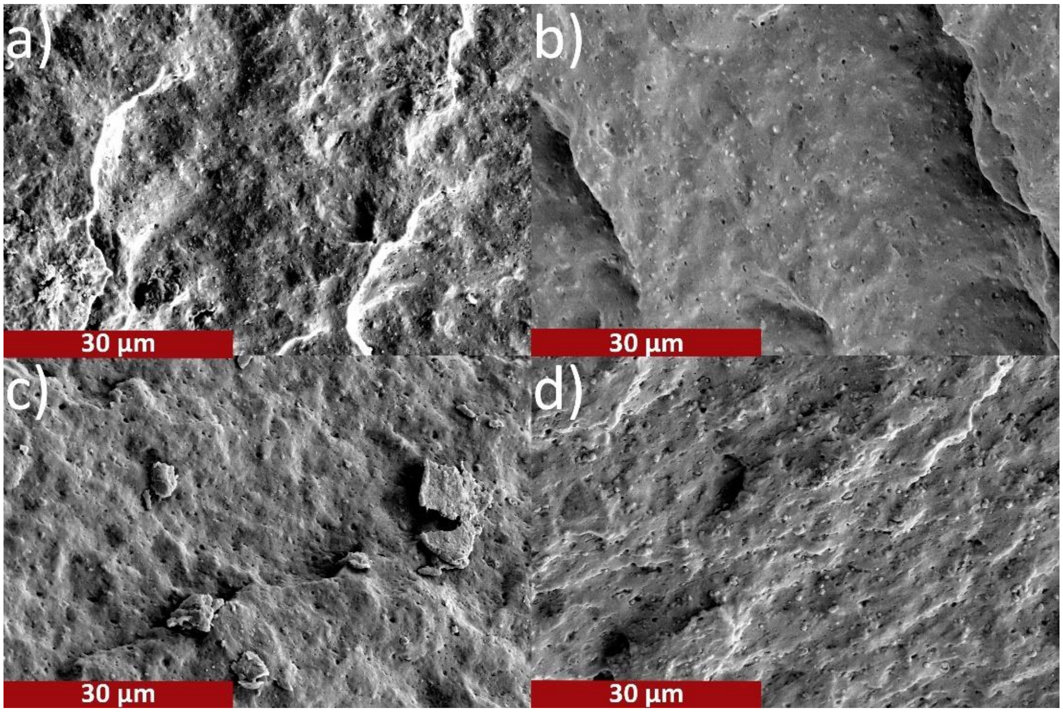

Figure S2. Scanning electron micrographs of cryo-fractured PHB and its blends with Terratek FX1515. a) PHB; b) 10F-PHB; c) 20F-PHB; and d) 30F-PHB.

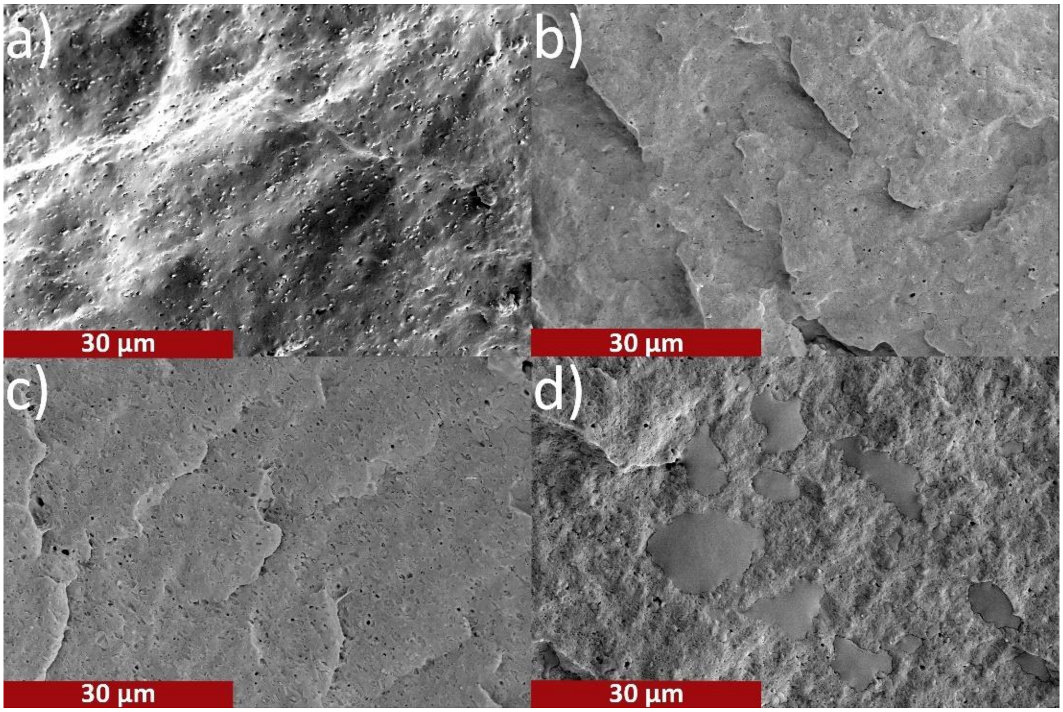

Figure S3. Scanning electron micrographs of impact-fractured PHB and its blends with Terratek FX1515. a) PHB; b) 10F-PHB; c) 20F-PHB; and d) 30F-PHB.

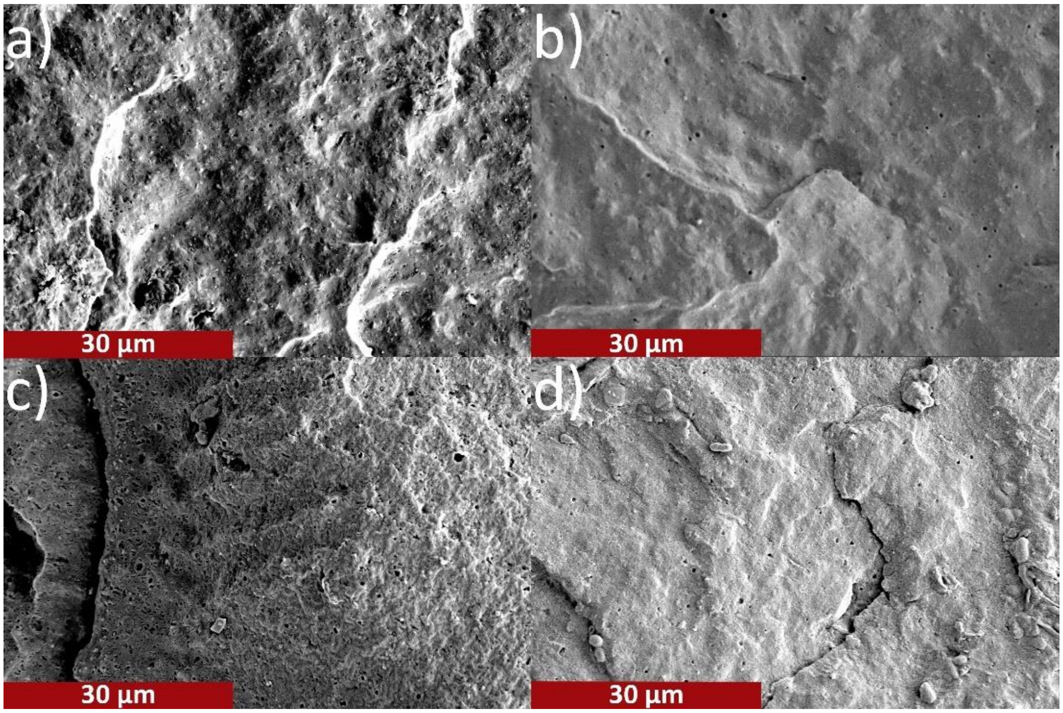

Figure S4. Scanning electron micrographs of cryo-fractured PHB and its blends with Terratek GDH-B1FA. a) PHB; b) 10G-PHB; c) 20G-PHB; and d) 30G-PHB.

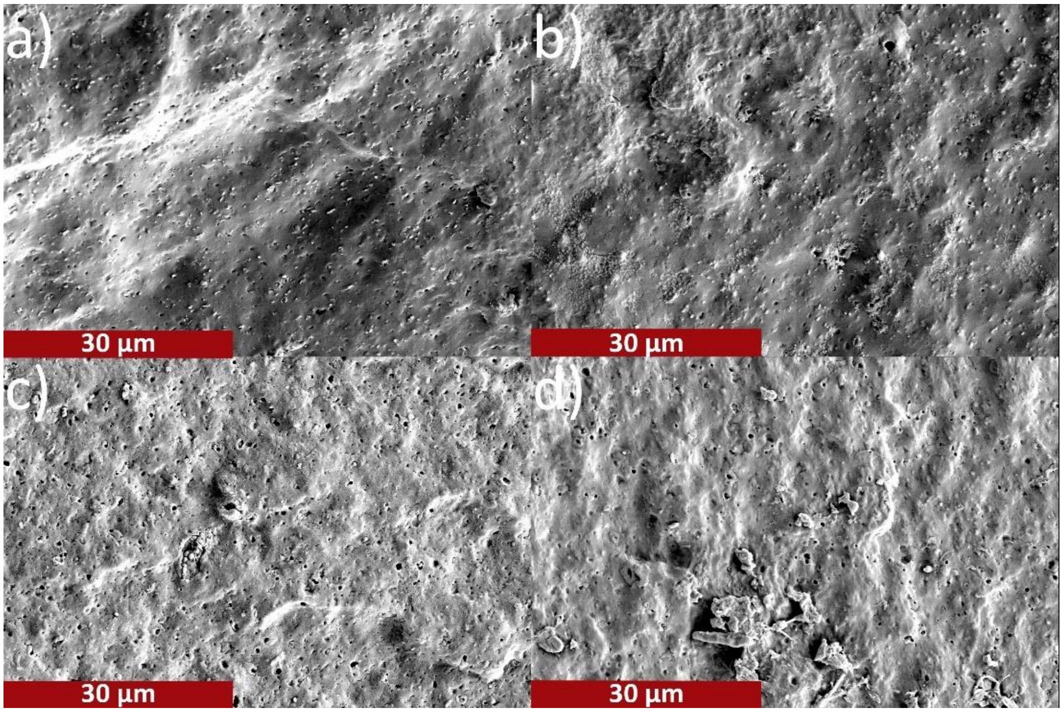

Figure S5. Scanning electron micrographs of impact-fractured PHB and its blends with Terratek GDH-B1FA. a) PHB; b) 10G-PHB; c) 20G-PHB; and d) 30G-PHB.

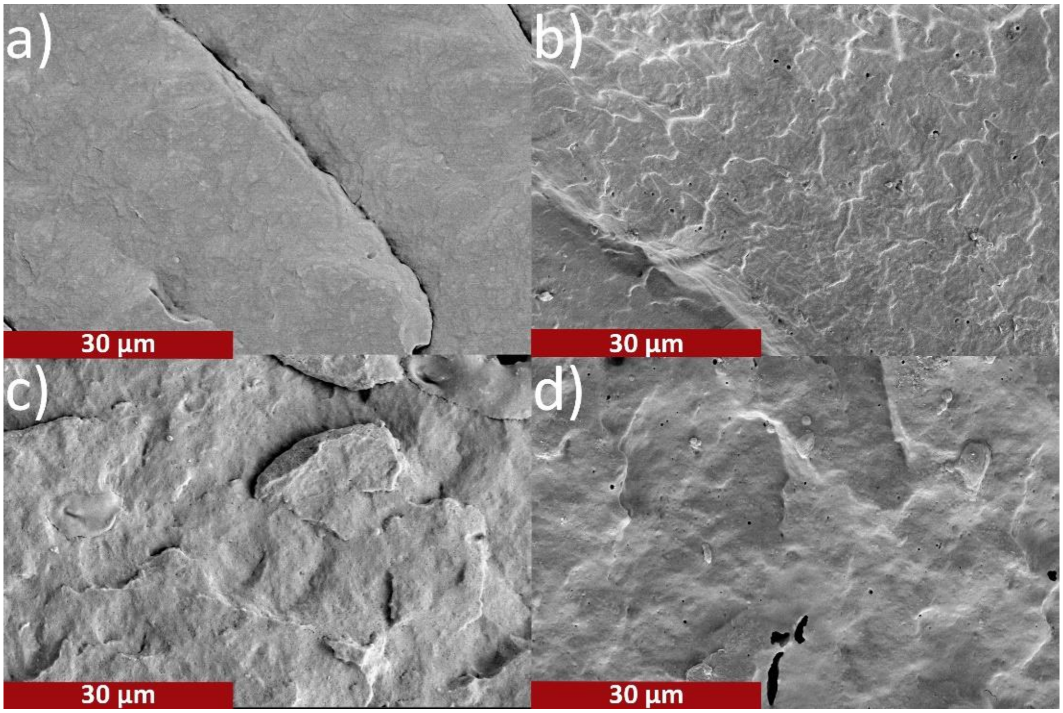

Figure S6. Scanning electron micrographs of cryo-fractured PHB-*co*-HHx and its blends with Terratek FX1515. a) PHB-*co*-HHx; b) 10F-PHB-*co*-HHx; c) 20F-PHB-*co*-HHx; and d) 30F-PHB-*co*-HHx.

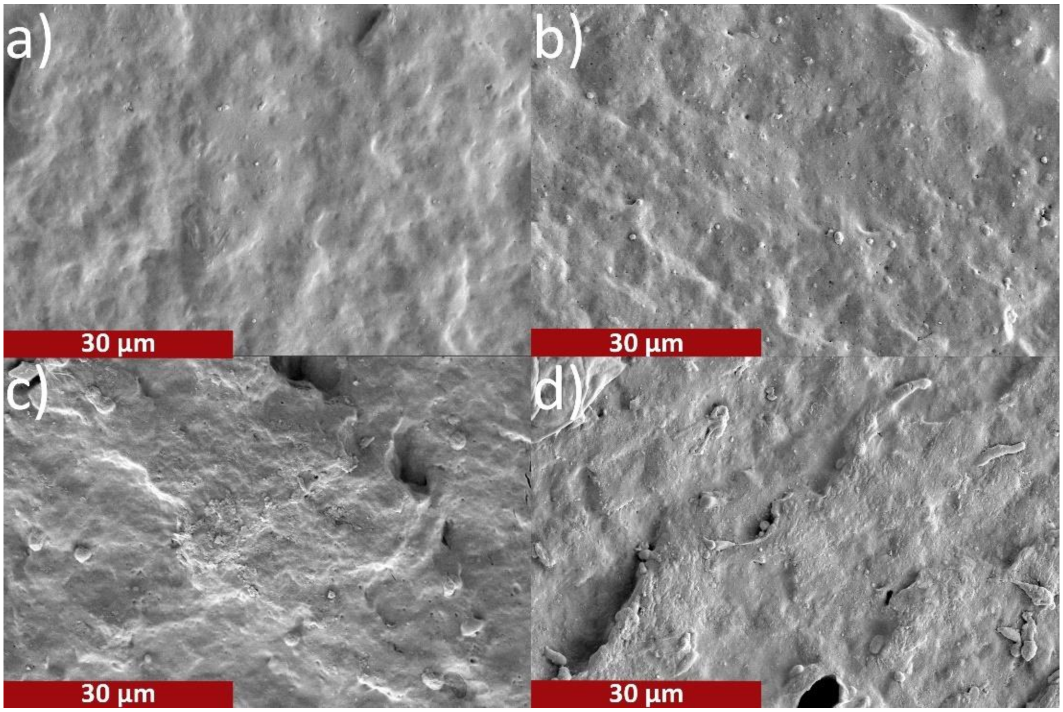

Figure S7. Scanning electron micrographs of impact-fractured PHB-*co*-HHx and its blends with Terratek FX1515. a) PHB-*co*-HHx; b) 10F-PHB-*co*-HHx; c) 20F-PHB-*co*-HHx; and d) 30F-PHB-*co*-HHx.

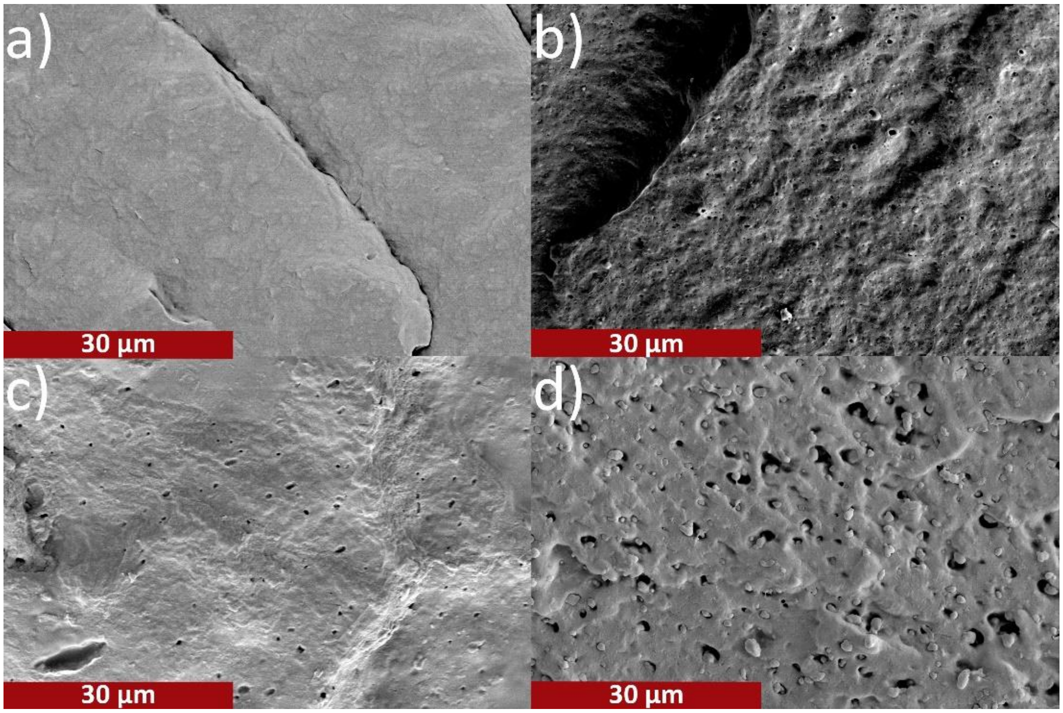

Figure S8. Scanning electron micrographs of cryo-fractured PHB-*co*-HHx and its blends with Terratek GDH-B1FA. a) PHB-*co*-HHx; b) 10G-PHB-*co*-HHx; c) 20G-PHB-*co*-HHx; and d) 30G-PHB-*co*-HHx.

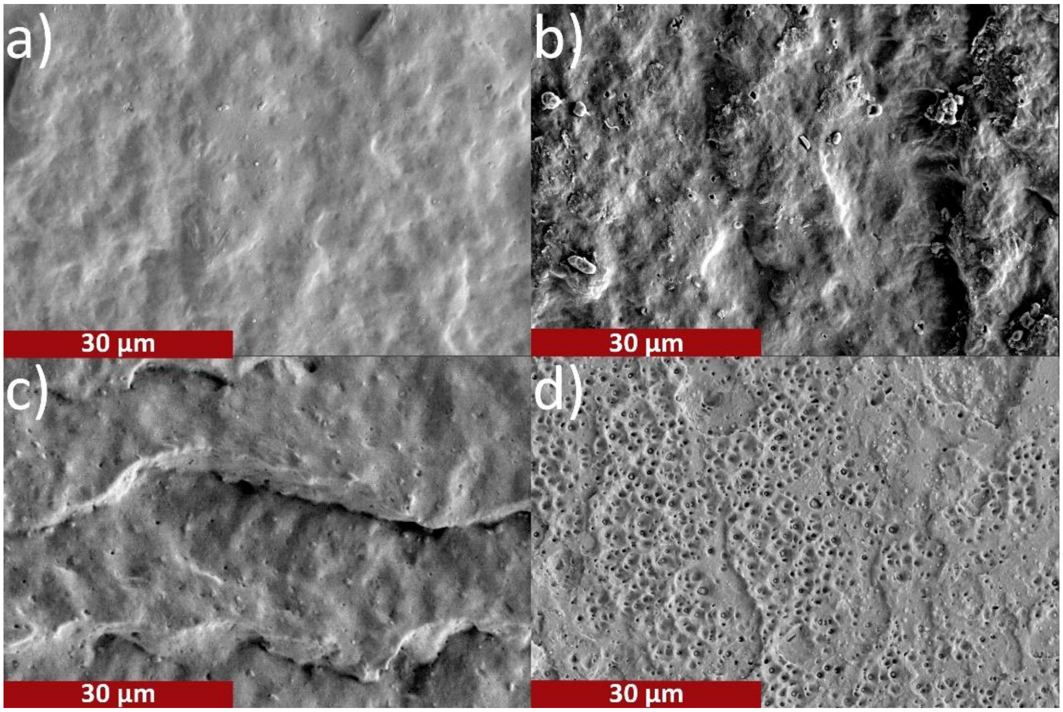

Figure S9. Scanning electron micrographs of impact- PHB-*co*-HHx and its blends with Terratek GDH-B1FA. a) PHB-*co*-HHx; b) 10G-PHB-*co*-HHx; c) 20G-PHB-*co*-HHx; and d) 30G-PHB-*co*-HHx.

Dynamic Mechanical Analysis

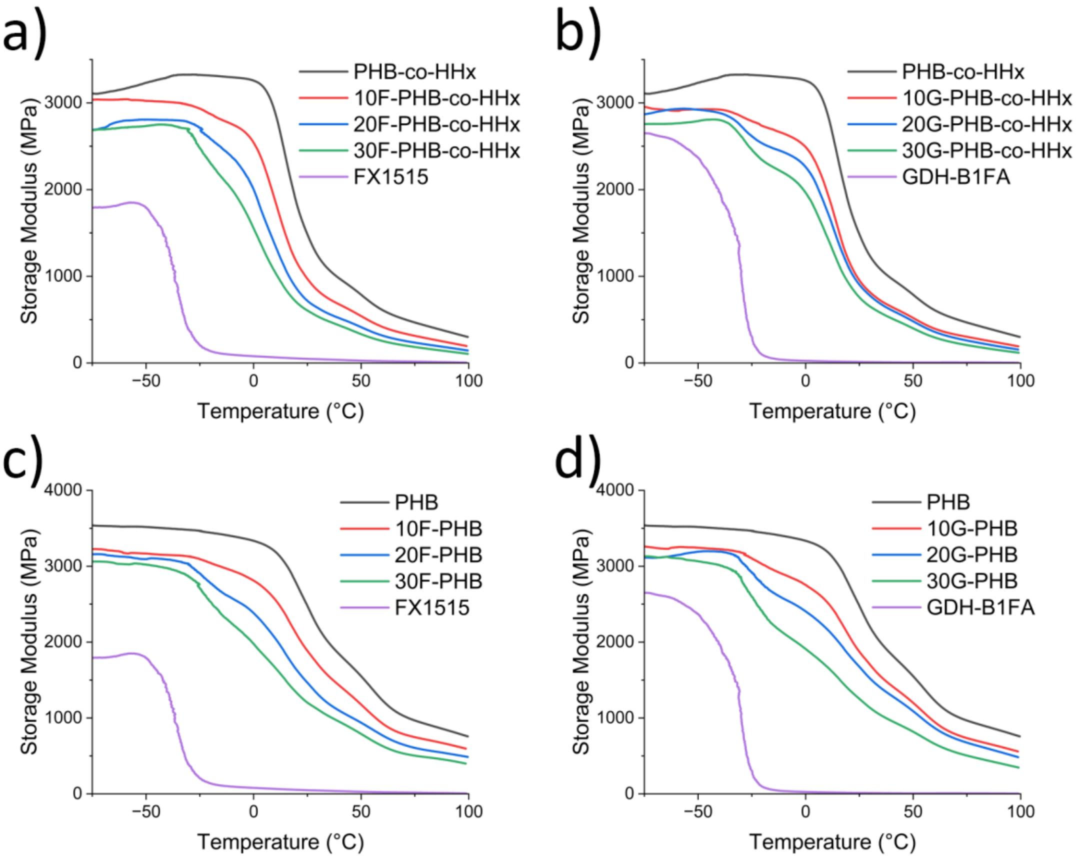

Figure S10. Storage modulus thermograms of PHAs, Terratek resins and their blends. a) F-PHB-*co*-HHx; b) G-PHB-*co*-HHx; c) F-PHB; and d) G-PHB.

Surface Energy

Table S2. Surface energy of the solvents which were used for contact angle measurement.

Liquid	Surface Energy (mN/m)	Dispersive Component (mN/m)	Polar Component (mN/m)
Water	72.8	26.4	46.4
Water:Ethylene glycol (1:1 v/v%)	57.7	20	37.9
Ethylene glycol	47.7	26.4	21.3
Benzyl alcohol	39.0	30.3	8.7
Cyclohexane	25.5	25.5	0

Table S3. Contact angle measurements of PHAs and impact modifiers with different solvent systems.

Sample	Cyclohexane	Benzyl Alcohol	Ethylene Glycol	Ethylene Glycol/Water (1:1)	Water
PHB	8.5 ± 4.6	15.2 ± 4.0	45.5 ± 4.2	60.6 ± 1.7	65.7 ± 1.0
PHB-*co*-HHx	35.7 ± 8.0	4.2 ± 1.0	51.4 ± 1.2	72.3 ± 2.2	75.7 ± 4.3
FX1515	7.9 ± 1.2	17.7 ± 5.1	35.3 ± 1.9	51.4 ± 2.7	57.6 ± 1.9
GDH-B1FA	8.8 ± 3.2	48.0 ± 4.0	57.9 ± 2.7	67.4 ± 1.65	71.5 ± 2.6

Table S4. Surface energy of PHAs and Impact modifiers calculated using the Owens, Wendt, Rabel and Kaelble (OWRK) model.

Sample	Surface Energy (mN/m)	Dispersive Component (mN/m)	Polar Component (mN/m)
PHB	36.59	25.51	11.08
PHB-*co*-HHx	32.56	24.64	7.92
FX1515	41.47	23.44	18.04
GDH-B1FA	31.61	21.57	7.9

Biodegradation Studies

Respirometry methods and Calculations

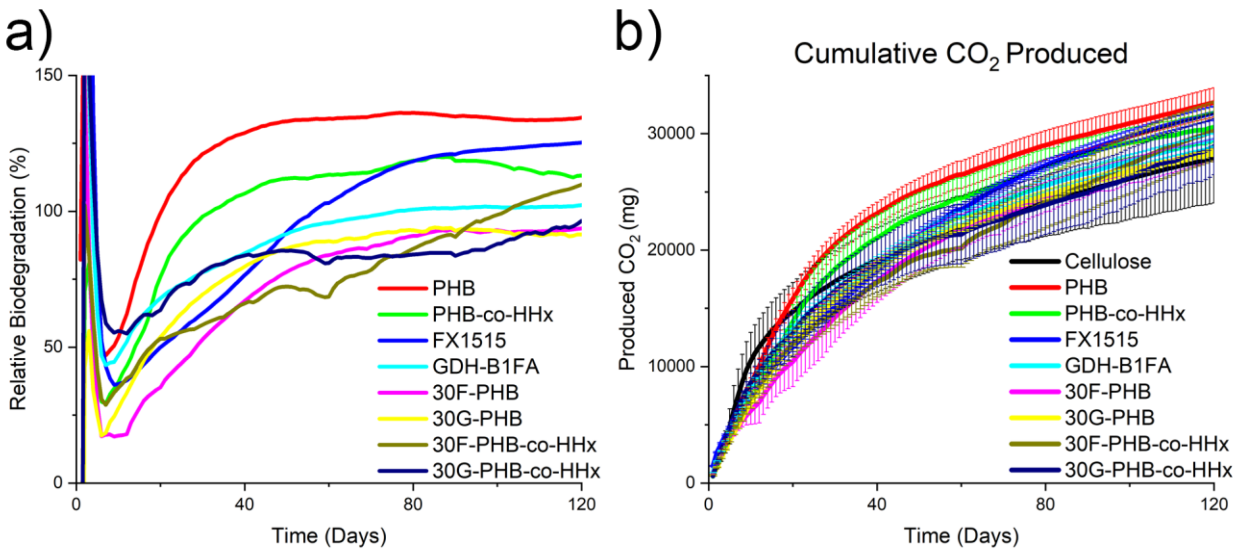

Figure S11. Respirometry study results. a) Biodegradation of PHAs, Terratek resins, and the 30 w/w% loadings of impact modifiers in PHAs relative to the positive control cellulose. b) Cumulative CO_2 evolution.

Polymer degradation was determined by gas evolution and monitored using an ECHO instruments respirometer (SI). The sample CO_2 production (s) values were calculated daily by taking the difference in CO_2 production (in mg) of each reactor (r) and the daily average CO_2 production from blank controls (b) as described by equation S1.

Equation S1: $$s = r - b$$

Using the sample mass (m) and the percent organic carbon content (C), and the dimensionless term of 44.01/12.01, the absolute biodegradation values were calculated (equation S2). The dimensionless term accounts for the carbon mass contribution from the CO_2 generated from each reactor for each day.

Equation S2: $$absolute\ biodegradation\ (\%) = \frac{s}{mc\left(\frac{44.01}{12.01}\right)}$$

The organic carbon and nitrogen were analyzed with an organic elemental analyzer using the combustion method and TCD detection. The measured elemental composition of the compost was 19.46% carbon and 1.73% nitrogen (C/N = 11.25).

Table S5. Total carbon and nitrogen elemental analysis, ammonium and nitrate analysis, and lime buffer capacity (LBC) results.

**Sample**	**LBC (ppm $CaCO_3$/pH)**	**pH**	**NH_4-N**	**NO_3-N**	**C**	**N**
Compost	617	7.66	1.72	436.4	19.46	1.73

Table S6. Final biodegradation values reached by each sample at the end of testing at 120 days.

**Sample**	**Cumulative CO_2 (mg)**	**Absolute Biodegradation (%)**	**Relative Biodegradation (%)**
Cellulose	27878.9 ± 3843.1	90.8	n/a
PHB	32679.8 ± 1249.9	122.0	134.4
PHB-*co*-HHx	30556.1 ± 1311.3	102.7	113.2
FX1515	31768.4 ± 583.9	113.7	125.3
GDH-B1FA	29468.7 ± 559.6	92.8	102.3
30F-PHB	28610.8 ± 938.5	85.0	93.7
30G-PHB	28391.9 ± 297.2	83.0	91.5
30F-PHB-*co*-HHx	30219.3 ± 2523.5	99.6	109.8
30G-PHB-*co*-HHx	28889.2 ± 2404.5	87.0	96.5
